# Differential expression of immune activation markers in T cells from patients with multiple myeloma

**DOI:** 10.3389/fonc.2026.1788729

**Published:** 2026-05-20

**Authors:** Jiaoya Lin, Xin Zhang, Yifei Zhao, Lan Luo, Hong Yuan, Ling Zhong

**Affiliations:** 1 Department of Clinical Laboratory Diagnosis, The Affiliated Hospital, Southwest Medical University, Luzhou, Sichuan, China; 2 Second Clinical School, Tongji Medical College, Huazhong University of Science and Technology, Wuhan, China; 3 Department of Clinical Laboratory Diagnosis, Sichuan Provincial People’s Hospital, Chengdu, Sichuan, China

**Keywords:** multiple myeloma, tumor microenvironment, CD4^+^ T cell, CD8^+^ T cell, CD95, CD278, HLA-DR

## Abstract

**Introduction:**

Multiple myeloma (MM) is the second most common hematological malignancy, characterized by the abnormal proliferation of malignant plasma cells leading to the classic CRAB symptoms. Despite advances in treatment, MM remains incurable. Alterations in the tumor immune microenvironment (TIME) contribute to therapy resistance. However, the activation status of T cells, key regulators of immune responses, is still not fully understood in MM.

**Methods:**

To further investigate immune alterations in the tumor microenvironment, bone marrow samples were collected from 18 newly diagnosed MM patients and 12 age- and sex-matched healthy controls. Multiparameter flow cytometry was used to analyze T cell subsets and their activation markers.

**Results:**

We observed a significant increase in the proportions of CD4^+^ central memory T cells (Tcm) and CD8^+^ Tcm in MM patients, while CD4^+^ effector memory T cells (Tem) and CD4^+^ TEMRA cells were significantly decreased. In terms of activation markers, MM patients showed increased frequencies of CD95^+^ CD4^-^ TEMRA, CD95^+^ CD8^-^ Tn, CD278^+^ CD4^-^ TEMRA, and HLA-DR^+^ CD4^-^ Tem subsets compared to healthy controls. In contrast, CD95^+^ CD8^+^ Tcm, CD278^+^ CD8^+^ Tn, CD278^+^ CD8^+^ Tcm, and HLA-DR^+^ CD8^+^ Tcm subsets were significantly reduced.

**Discussion:**

These findings reveal differences in the composition and activation profiles of T cell subsets in the bone marrow of MM patients. While they provide insights into the immune landscape of MM, further studies are needed to clarify their functional significance and potential implications for therapeutic strategies.

## 1 Background

Multiple myeloma (MM) is the second most common hematologic malignancy after lymphoma, accounting for approximately 10% to 15% of all hematologic cancers (1). It is characterized by the clonal proliferation of malignant plasma cells in the bone marrow, which disrupts normal hematopoiesis and leads to a range of clinical manifestations, including hypercalcemia, renal impairment, anemia, and bone lesions. Current research widely acknowledges that the proliferation and survival of MM cells are strongly influenced by the bone marrow microenvironment, which plays a critical role in promoting immune evasion and the development of drug resistance (2). The bone marrow microenvironment is a complex network composed of various stromal and cellular components, including mesenchymal stem cells, osteoblasts, osteoclasts, endothelial cells, and immune cells such as T cells, natural killer (NK) cells, monocytes, and myeloid-derived suppressor cells (MDSCs) (3, 4). The activation status of immune cells is central to the progression of MM, as dysregulated immune responses can facilitate immune escape. For instance, elevated levels of interleukin-6 (IL-6) and transforming growth factor-β (TGF-β) in MM patients can reduce the number of CD4^+^ T cells, disrupt the Th1/Th2 balance, promote the expansion of Th17 cells, and ultimately impair immune surveillance (5). Previous studies have shown that during the transition from monoclonal gammopathy of undetermined significance (MGUS) to overt MM, the numbers of CD4^+^ and CD8^+^ T cells markedly decline, while terminally differentiated CD8^+^ effector T cells and CD14^+^CD16^+^ monocytes significantly increase (6, 7). Although T cell-engaging therapies (TCEs) can partially overcome the reduction in T cell numbers, their efficacy largely depends on the functional status of the remaining T cells. In cases of severe immune exhaustion, the clinical benefit of TCEs is often limited. Therefore, a better understanding of T cell immune competence in MM patients is essential for elucidating disease mechanisms and optimizing immunotherapeutic strategies (8). Previous studies (9) have demonstrated that distinct subsets of CD4^+^ cytotoxic T lymphocytes (CTLs) are significantly increased in MM patients and exhibit high expression of NKG2D. The expansion of this CD4^+^ CTL subset is positively correlated with patient survival. Moreover, the abundance of CD4^+^ T cells *in vivo* is associated with the durability of the response to B-cell maturation antigen (BCMA)-targeted chimeric antigen receptor (CAR) T-cell therapy in relapsed and refractory MM (10). Similarly, a study (11) on immune responses and non-responses in CAR-T therapy for relapsed and refractory patients found that CD8^+^ T cells and NK cells in non-responders were in an immunosuppressive state, revealing that the immunosuppressive microenvironment is one of the reasons for resistance to CAR-T cell therapy in MM patients. In the T cell MYC immune-driven transgenic mouse model, MM progression regulated immune evasion mechanisms and reshaped the bone marrow microenvironment in distinct ways. A large number of exhausted and activated CD8^+^ T cells appeared, while regulatory T cells (Tregs) significantly decreased. By increasing the cytotoxicity of CD8^+^ T cells, long-term suppression of MM could be achieved (12). Therefore, the activation of T cells is crucial for the treatment of tumors.

CD95 is a dual-function receptor that plays a crucial role in fine-tuning primary T cell activation, exerting pro-apoptotic or anti-apoptotic effects depending on the cellular environment, activation status, and ligand binding mode (13). In patient MM cells, the use of alkyl-lysophospholipid drugs can induce the activation of the CD95 death receptor, leading to the formation of the death-inducing signaling complex, which further triggers cell apoptosis (14). The Apo-1/CD95 ligand on T cells can protect effector T cells from being killed by myeloma cells while performing immune functions (15). This suggests that CD95 plays a protective role in T cells. However, there is currently limited research on the role of CD95 in T cell subsets in multiple myeloma.

Inducible T-cell co-stimulator (ICOS, CD278) is an activating co-stimulatory immune checkpoint expressed on activated T cells. Its ligand is expressed on both tumor cells and antigen-presenting cells. Upon binding to its ligand, ICOS elicits diverse functions in T cells, including immune activation and effector responses (16). Azithromycin (AZM), a macrolide antibiotic, suppresses the immune function of CD4^+^ T cells by downregulating the expression of CD278 (ICOS) on their surface (17). At present, research on CD278 within the tumor microenvironment and tumor cells of multiple myeloma remains limited. Therefore, elucidating its role in T cells is crucial for understanding the pathogenesis and progression of multiple myeloma.

HLA-DR, a major histocompatibility complex class II (MHC-II) molecule, plays a pivotal role in the adaptive immune response by presenting extracellular antigens to CD4^+^ T helper cells. It is primarily expressed on professional antigen-presenting cells (APCs), including dendritic cells, monocytes/macrophages, and B cells. Previous studies have demonstrated that significant alterations in the tumor immune microenvironment (TME) occur at early stages of disease progression, from MGUS to smoldering multiple myeloma (SMM). Notably, a marked downregulation of HLA-DR expression has been observed on CD16^+^ monocytes and plasmacytoid dendritic cells (18), suggesting that HLA-DR may serve as a potential diagnostic marker for clinical progression in multiple myeloma. The increased expression of HLA-DR in plasma cells and the tumor microenvironment is associated with a significant enhancement in CD4^+^ and CD8^+^ T cell infiltration, reflecting a more inflamed immune microenvironment (19). However, the role of HLA-DR expression within CD4^+^ and CD8^+^ T cell subsets in multiple myeloma remains largely unexplored, which is crucial for advancing the development of effective immunotherapeutic strategies for MM.

In this study, we collected clinical samples from patients with multiple myeloma and analyzed the changes in CD4^+^ and CD8^+^ T cell subsets under real-world conditions using a well-established flow cytometry panel (20). Furthermore, we investigated the expression patterns of activation markers CD95, CD278 (ICOS), and HLA-DR within each T cell subset. By characterizing the distribution of these markers, our findings may provide clinical guidance for selecting therapeutic strategies tailored to different immune microenvironments, to minimize drug resistance and prolong progression-free survival in patients.

## 2 Materials and methods

### 2.1 Subject information and clinical sample collection

We collected bone marrow samples from 18 patients with MM and 12 healthy controls, whose samples were obtained from bone marrow donors free of infection and hematologic diseases and who met the health criteria for bone marrow donation, and subjected all samples to flow cytometry analysis. The median age of the MM group was 61 years (range: 55–82 years), while the median age of the healthy control group was 54 years (range: 45–80 years), with no significant age difference between the two groups. The MM group consisted of 10 male and 8 female patients. According to the Revised International Staging System (R-ISS), 1 patient was in stage I, 6 patients were in stage II, and 11 patients were in stage III. Bone marrow samples were obtained through aspiration as part of the diagnostic procedure, performed by clinicians during routine examination. Approximately 2–3 mL of bone marrow was collected per aspiration, and samples were immediately placed into EDTA tubes for preservation. All samples underwent flow cytometric analysis within 12 hours of collection.

### 2.2 Collection of blood samples and subsequent fluorochrome staining

A 5 mL bone marrow sample was collected from each subject into EDTA-coated tubes. The sample was transferred to a 10 mL centrifuge tube and brought to a final volume of 10 mL with sterile, enzyme-free phosphate-buffered saline (PBS). The suspension was centrifuged at 450 × g for 5 minutes, and the supernatant was discarded. The washing step was repeated three times with PBS. After the final wash, a small volume of supernatant and the entire cell pellet were retained.

A 100 μL aliquot of the washed bone marrow cell suspension was transferred to a flow cytometry tube, followed by the addition of 10μL of each T-cell surface monoclonal antibody. The antibody panel included the following:CD95-FITC (Cat# 335099, BioLegend),CD197-PE (Cat# B07621, Beckman Coulter),HLA-DR-ECD (Cat# B50149, Beckman Coulter),CD45RA-PC7 (Cat# B10821, Beckman Coulter),CD278-APC (Cat# 313509, BioLegend),CD3-AA700 (Cat# B10823, Beckman Coulter),CD127-AA750 (Cat# B12700, Beckman Coulter),CD4-PB (Cat# B49197, Beckman Coulter),CD8-KRO(Cat# B00067, Beckman Coulter).The mixture was vortexed gently and incubated for 15 minutes at room temperature in the dark. After incubation, 500 μL of OptiLyse No-Wash Lysing Solution (Beckman Coulter) was added to each tube, gently mixed, and further incubated for 10 minutes at room temperature in the dark to lyse red blood cells. Subsequently, PBS was added to bring the volume to 3 mL, followed by centrifugation at 450 × g for 5 minutes. The supernatant was discarded, and the cell pellet was resuspended in 300μL of PBS. Samples were then analyzed by flow cytometry.

### 2.3 Flow cytometric analysis

Multicolor flow cytometric analysis was performed using the Navios multiparameter flow cytometer (Beckman Coulter, USA). For each experiment, unstained samples were included as negative controls to ensure the accuracy of subsequent analyses. Flow cytometry data were analyzed using FlowJo software (version 10). Initially, lymphocyte populations were gated based on forward scatter (FSC) and side scatter (SSC) parameters. To exclude cell doublets, a FSC-A versus FSC-H plot was applied, ensuring that only single cells were included in the analysis. CD3^+^ T cells were identified by gating on CD3-AA700 versus SSC, and this population was subsequently used as the parent gate for further analysis. Within the CD3^+^ T cell population, CD4^+^ and CD8^+^ T cell subsets were distinguished using CD4-PB and CD8-KRO markers. Further gating within the CD4^+^ or CD8^+^ T cell populations was performed using CD45RA and CD197 to define T cell subsets, including naïve T cells (Tn), central memory T cells (Tcm), effector memory T cells (Tem), and terminally differentiated effector memory T cells re-expressing CD45RA (TEMRA). Finally, the expression frequencies of CD95, CD278, and HLA-DR were analyzed within each T cell subset. All results were exported using the Table Editor function in FlowJo for further statistical analysis and data presentation.

### 2.4 Statistical analysis

Statistical analysis was performed using SPSS software (version 27.0) based on data exported from FlowJo. Patient group information and variable names corresponding to various detection indicators were first entered into the variable view of SPSS, and the data were organized accordingly. Normality of each group was assessed using the “Analyze – Descriptive Statistics – Explore” function. If the Shapiro-Wilk test yielded a p-value greater than 0.05, the data were considered to follow a normal distribution, and an independent samples t-test was conducted for group comparisons. Levene’s test for equality of variances was used to determine whether equal variances could be assumed: if *P* > 0.05, equal variances were assumed; if *P* < 0.05, equal variances were not assumed. For data that did not follow a normal distribution (Shapiro-Wilk test *P* ≤ 0.05), the Mann–Whitney U test was used to compare the medians between the two groups. P values were adjusted using the Bonferroni correction. After correction, P values less than 0.05 were considered to indicate robust statistical significance, while unadjusted P values below 0.05 were interpreted as suggestive of statistical significance. Spearman correlation analysis was performed to examine the relationship between T-cell subset proportions and activation marker expression across different R-ISS stages. Since there was only one patient in stage I, this sample was combined with stage II for the analysis.

## 3 Results

### 3.1 Analysis of the changes in the proportions of CD4^+^ and CD8^+^ T cell subsets in the bone marrow of multiple myeloma patients

**Figure 1:**
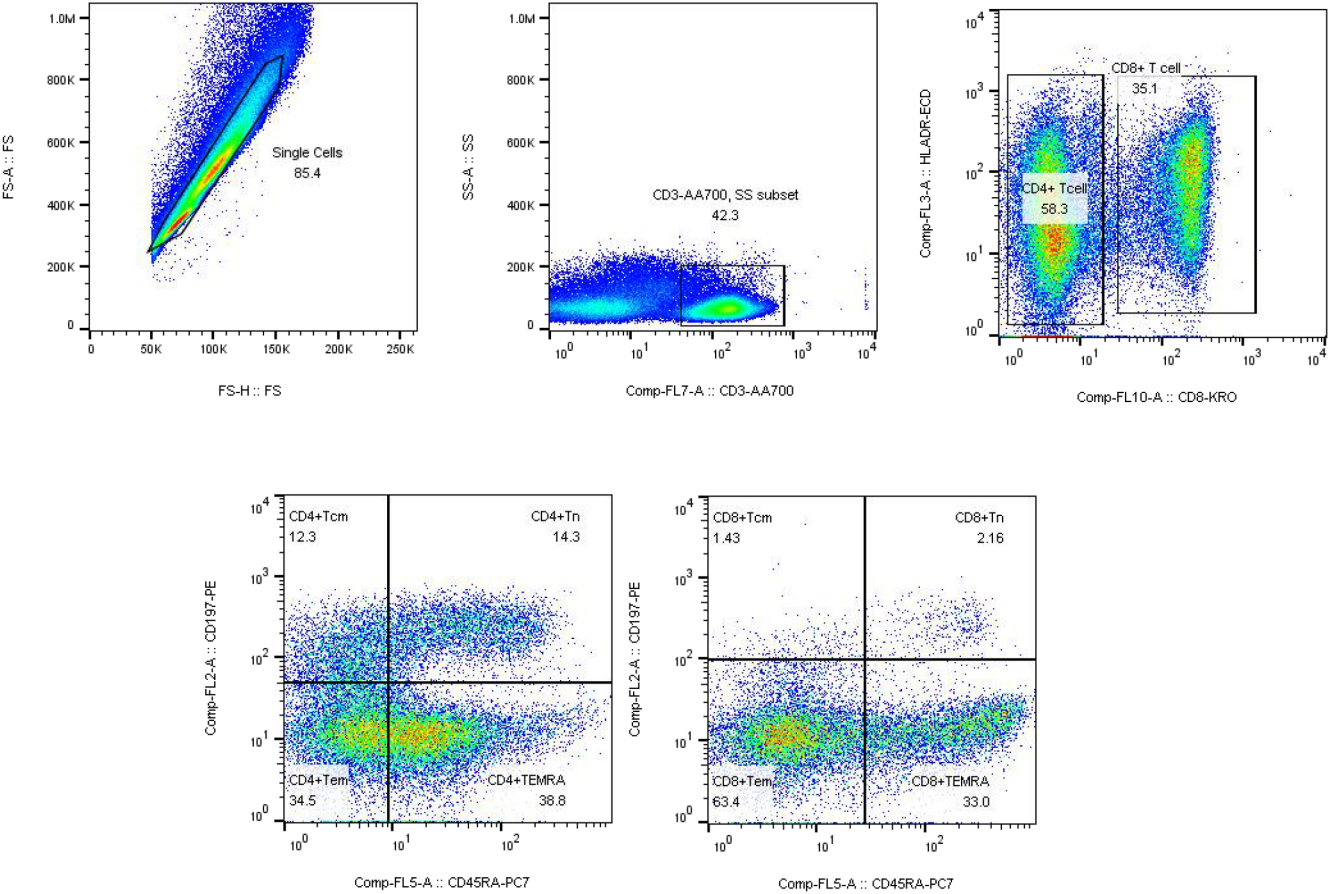
Identification of CD4^+^ and CD8^+^ T cells in bone marrow. The flow cytometric gating strategy used to identify CD4^+^ and CD8^+^ T-cell subsets in bone marrow samples from patients with multiple myeloma and healthy donors is shown.

We first examined the correlation between different T-cell subsets and R-ISS stages. No significant clinical association or statistical difference was observed among the stages (**Supplementary Tables 1**, **2**). Therefore, patients with different R-ISS stages were combined into a single multiple myeloma (MM) group for subsequent analyses. Comparative analysis of CD4^+^ and CD8^+^ T cell subsets in the bone marrow of patients with MM and healthy controls revealed significant differences between the two groups. In MM patients, the proportion of CD4^+^ central memory T cells (Tcm, CD45RA^-^CD197^+^) among total CD4^+^ T cells was significantly elevated (50.03 ± 14.34%) compared to that in healthy individuals (14.11 ± 25.90%) (P < 0.001). Conversely, the proportion of CD4^+^ effector memory T cells (Tem, CD45RA^-^CD197^-^) was markedly reduced in MM patients (33.78 ± 9.63%) relative to controls (58.99 ± 23.96%) (P = 0.004). Similarly, CD4^+^ TEMRA cells (CD45RA^+^CD197^-^) were significantly decreased in MM patients (2.47 ± 8.28%) compared to the healthy group (12.07 ± 9.94%) (P = 0.005). Among CD8^+^ T cell subsets, only the proportion of CD8^+^ Tcm cells (CD45RA^-^CD197^+^) was significantly higher in MM patients (7.38 ± 6.85%) than in controls (2.77 ± 5.25%) (P < 0.001) while no significant differences were observed in other CD8^+^ T cell subsets between the two groups (**Table 1**) (**Figure 1**). Collectively, these results indicate a marked increase in CD4^+^Tcm and CD8^+^Tcm cells, accompanied by a significant reduction in CD4^+^Tem and CD4^+^TEMRA cells in the bone marrow of patients with multiple myeloma.

**Table 1:** Proportions of CD4^+^ and CD8^+^ T cell subsets in the bone marrow of multiple myeloma patients and healthy controls.

Cell cluster	MM (%)	Normal (%)	*P*-value	Adjusted *P*
CD4^+^Tn(CD45RA^+^CD197^+^)	13.72 ± 9.68	14.82 ± 13.76	0.983	1
CD4^+^Tcm(CD45RA-CD197^+^)	50.03 ± 14.34	14.11 ± 25.90	<0.001	0.0096
CD4^+^Tem(CD45RA-CD197-)	33.78 ± 9.63	58.99 ± 23.96	0.004	0.0652
CD4^+^TEMRA(CD45RA^+^CD197-)	2.47 ± 8.28	12.07 ± 9.94	0.005	0.0538
CD8^+^ Tn(CD45RA^+^CD197^+^)	5.38 ± 7.40	10.80 ± 15.22	0.917	1
CD8^+^Tcm(CD45RA-CD197^+^)	7.38 ± 6.85	2.77 ± 5.25	<0.001	0.01288
CD8^+^Tem(CD45RA-CD197-)	66.16 ± 15.31	61.43 ± 19.92	0.468	1
CD8^+^TEMRA(CD45RA^+^CD197-)	21.08 ± 14.27	25.00 ± 14.32	0.468	1

### 3.2 Analysis of CD95 expression in CD4^+^ and CD8^+^ T cell subsets in the bone marrow

**Figure 2:**
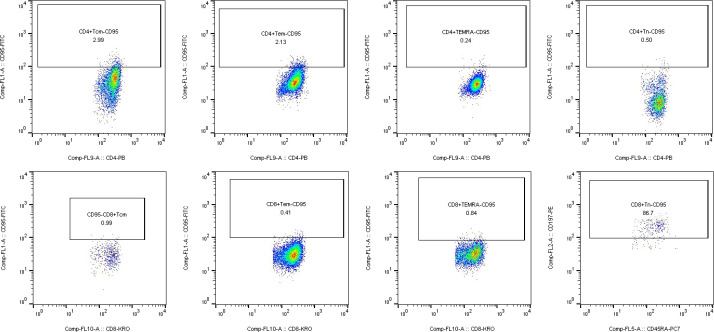
Gating strategy for profiling CD95 expression on T cell subsets. This figure illustrates the flow cytometric identification and CD95 expression profile across CD4^+^ and CD8^+^ T cell subsets: naive (Tn), central memory (Tcm), effector memory (Tem), and TEMRA cells.

To investigate the expression of CD95 in CD4^+^ and CD8^+^ T cell subsets, we performed flow cytometric analysis to compare its distribution in bone marrow samples from patients with multiple myeloma and healthy controls. The results showed that the proportion of CD95^+^ CD4^+^ TEMRA cells was significantly higher in multiple myeloma patients (19.49 ± 13.53%) compared to healthy controls (12.37 ± 18.63%) (P = 0.025). In the CD8^+^ T cell compartment, the frequency of CD95^+^ CD8^+^ Tn cells in patients were markedly elevated (75.65 ± 26.26%) relative to controls (13.67 ± 30.45%) (P < 0.001). Conversely, the proportion of CD95^+^ CD8^+^ Tcm cells was significantly reduced in the multiple myeloma group (2.59 ± 3.53%) compared to the control group (30.85 ± 30.27%) (P = 0.008) (**Table 2**) (**Figure 2**). In summary, CD95 expression in multiple myeloma patients was predominantly upregulated in the CD4^+^ TEMRA and CD8^+^ Tn subsets, whereas a notable downregulation was observed in the CD8^+^ Tcm population.

**Table 2:** Expression of activation markers CD95, CD278, and HLA-DR in CD4^+^ and CD8^+^ T subcluster cells in the bone marrow of multiple myeloma patients and healthy controls.

Cell cluster	MM (%)	Normal (%)	*P*-value	Adjusted *P*
CD95^+^CD4^+^Tn	0.98 ± 1.91	3.45 ± 10.51	0.465	1
CD95^+^CD4^+^Tcm	3.36 ± 3.30	9.17 ± 14.02	0.185	1
CD95^+^CD4^+^Tem	5.57 ± 3.71	6.57 ± 6.27	0.755	1
CD95^+^CD4^+^TEMRA	19.49 ± 13.53	12.37 ± 18.63	0.025	0.6
CD95^+^CD8^+^Tn	75.65 ± 26.26	13.67 ± 30.45	<0.001	0
CD95^+^CD8^+^Tcm	2.59 ± 3.53	30.85 ± 30.27	0.008	0.192
CD95^+^CD8^+^Tem	0.58 ± 0.43	1.09 ± 1.66	0.851	1
CD95^+^CD8^+^TEMRA	0.40 ± 0.37	0.34 ± 0.44	0.518	1
CD278^+^CD4^+^Tn	1.69 ± 3.84	3.75 ± 10.43	0.787	1
CD278^+^CD4^+^Tcm	5.83 ± 4.37	10.95 ± 12.16	0.391	1
CD278^+^CD4^+^Tem	7.21 ± 5.20	7.67 ± 4.96	0.851	1
CD278^+^CD4^+^TEMRA	5.63 ± 6.94	0.37 ± 0.74	0.031	0.744
CD278^+^CD8^+^Tn	32.93 ± 23.27	49.09 ± 19.56	0.031	0.744
CD278^+^CD8^+^Tcm	5.80 ± 7.87	19.47 ± 14.94	0.002	0.048
CD278^+^CD8^+^Tem	2.51 ± 2.89	2.14 ± 1.71	0.692	1
CD278^+^CD8^+^TEMRA	10.11 ± 5.20	10.35 ± 8.56	0.932	1
HLA-DR^+^CD4^+^Tn	3.21 ± 3.61	4.76 ± 11.78	0.104	1
HLA-DR ^+^CD4^+^Tcm	5.78 ± 3.37	9.99 ± 14.08	0.692	1
HLA-DR ^+^CD4^+^Tem	25.64 ± 10.75	15.78 ± 10.50	0.019	0.456
HLA-DR ^+^CD4^+^TEMRA	37.63 ± 24.04	22.92 ± 33.01	0.079	1
HLA-DR ^+^CD8^+^Tn	1.18 ± 1.99	7.12 ± 10.43	0.346	1
HLA-DR ^+^CD8^+^Tcm	3.09 ± 4.61	40.27 ± 35.28	0.015	0.36
HLA-DR ^+^CD8^+^Tem	35.14 ± 12.50	35.02 ± 19.46	0.984	1
HLA-DR ^+^CD8^+^TEMRA	59.60 ± 19.00	48.63 ± 24.87	0.232	1

### 3.3 Analysis of the expression of CD278 in CD4^+^ and CD8^+^ T cell subsets in the bone marrow

**Figure 3:**
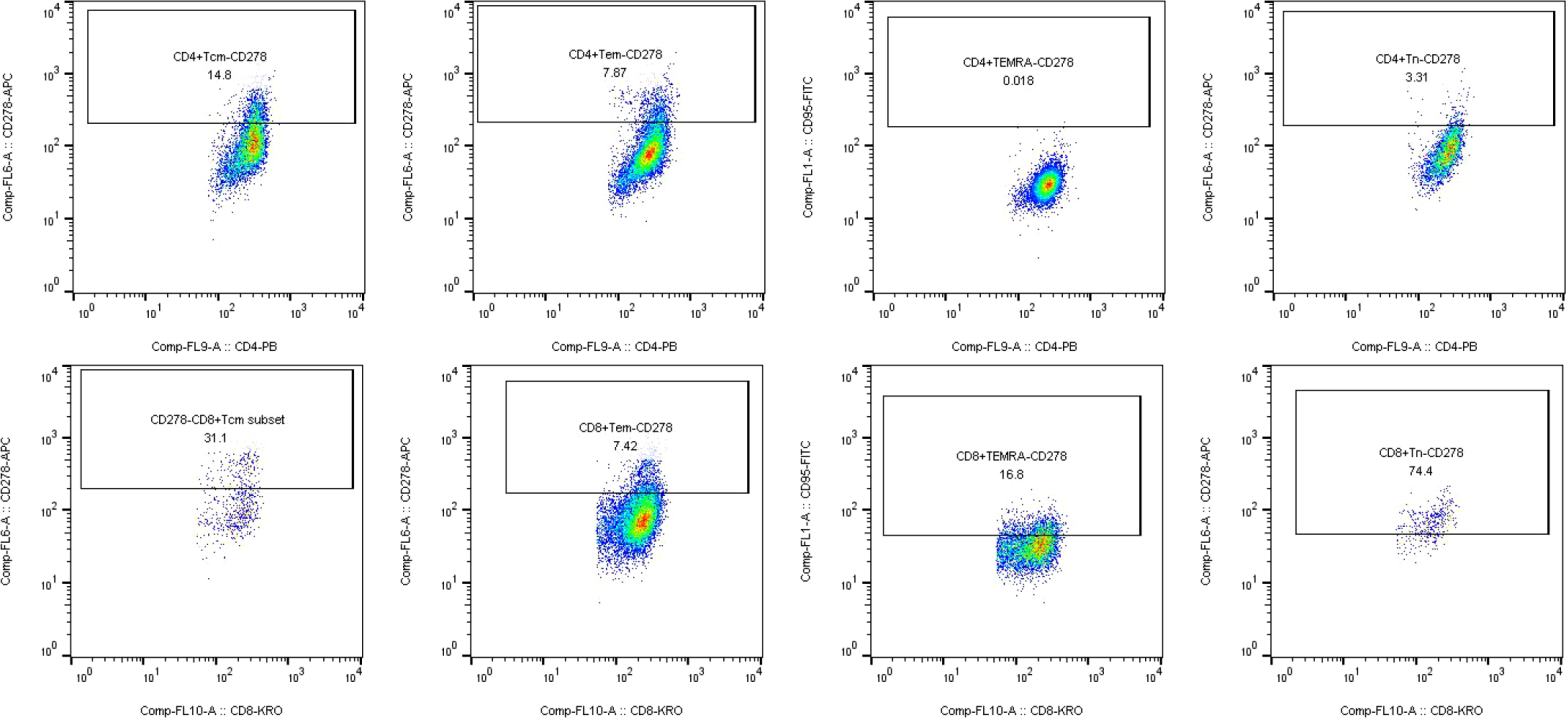
Profiling CD278 expression on T cell subsets. Gating strategy and expression profile of CD278 across CD4^+^ and CD8^+^ T cell subsets—including naive (Tn), central memory (Tcm), effector memory (Tem), and TEMRA cells—are shown.

Flow cytometric analysis of CD278 expression across various T cell subsets revealed significant alterations in patients with multiple myeloma. In the CD4^+^ T cell compartment, the proportion of CD278^+^ CD4^+^ TEMRA cells was markedly elevated in patients compared to healthy controls (5.63 ± 6.94% vs. 0.37 ± 0.74%, P = 0.031). Conversely, within the CD8^+^ T cell subsets, the frequency of CD278^+^ CD8^+^ Tn cells was significantly lower in the patient group than in the control group (32.93 ± 23.27% vs. 49.09 ± 19.56%, P = 0.031). Similarly, the percentage of CD278^+^ CD8^+^ Tcm cells was also significantly reduced in patients compared to healthy donors (5.80 ± 7.87% vs. 19.47 ± 14.94%, P = 0.002) (**Table 2**) (**Figure 3**). No statistically significant differences in CD278 expression were observed in other T cell subsets. Collectively, these findings suggest that CD278 exhibits subset-specific expression patterns in multiple myeloma, characterized by upregulation in CD4^+^ TEMRA cells and downregulation in CD8^+^ Tn and CD8^+^ Tcm cells.

### 3.4 Analysis of the expression of HLA-DR in CD4^+^ and CD8^+^ T cell subsets in the bone marrow

**Figure 4:**
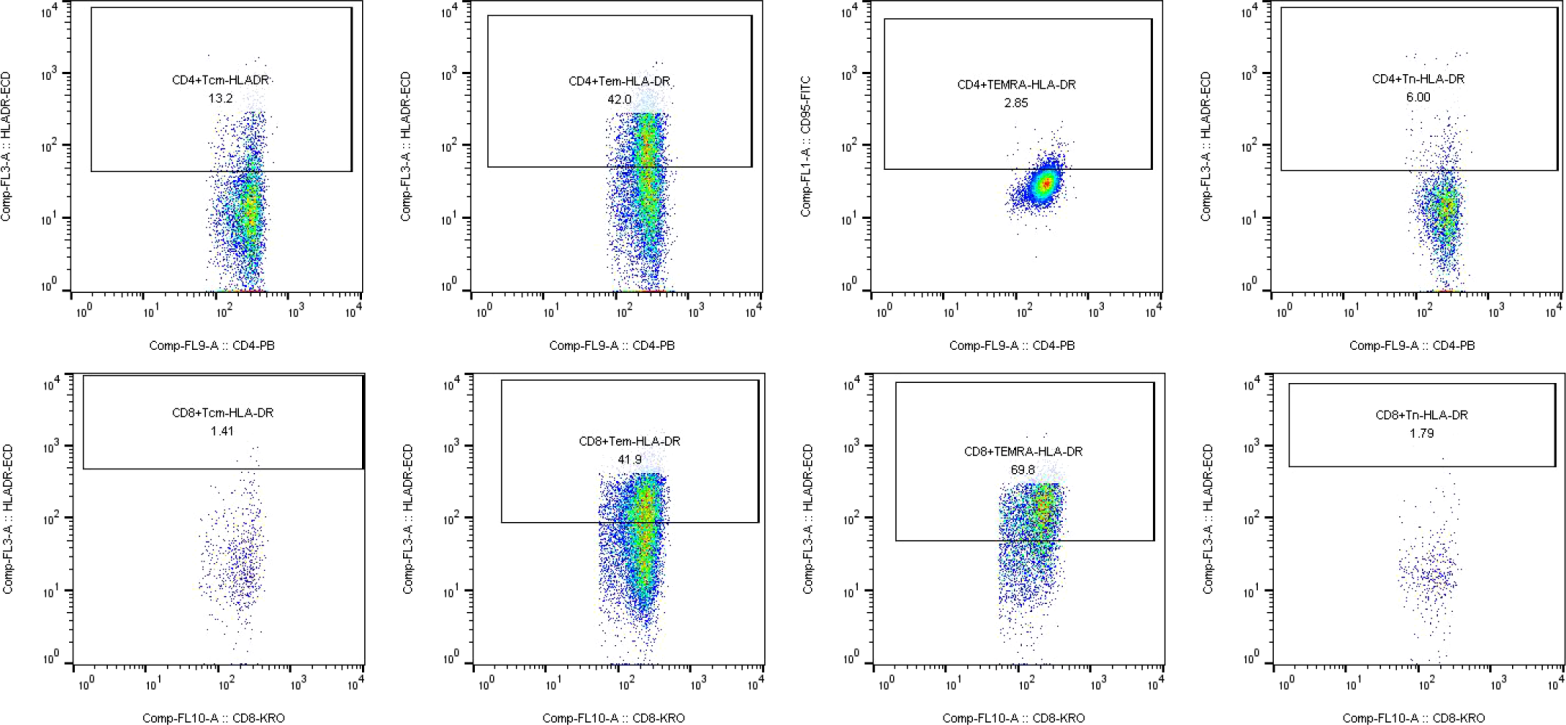
Gating strategy and profile of HLA-DR on T cell subsets. Analysis of HLA-DR expression across major CD4^+^ and CD8^+^ T cell subsets — naive (Tn), central memory (Tcm), effector memory (Tem), and TEMRA cells — is presented.

Analysis of HLA-DR expression across CD4^+^ and CD8^+^ T-cell subsets revealed significant differences in specific populations between multiple myeloma patients and healthy controls. Notably, the proportion of HLA-DR^+^ CD4^+^ Tem cells was significantly higher in multiple myeloma patients (25.64 ± 10.75%) compared to healthy individuals (15.78 ± 10.50%) (P = 0.019). In contrast, HLA-DR expression in CD8^+^ Tcm cells was significantly reduced in patients (3.09 ± 4.61%) relative to controls (40.27 ± 35.28%) (P = 0.015) (**Table 2**) (**Figure 4**). No significant differences in HLA-DR expression were observed among other T-cell subsets. In summary, HLA-DR expression is upregulated in CD4^+^ Tem cells and markedly downregulated in CD8^+^ Tcm cells in patients with multiple myeloma.

## 4 Discussion

In this study, we collected bone marrow samples from MM patients across different R-ISS stages and performed a comprehensive analysis of T cell subsets within the bone marrow immune microenvironment using multicolor flow cytometry. T cells were classified into eight subsets: CD4^+^ Tn, Tcm, Tem, TEMRA, and CD8^+^ Tn, Tcm, Tem, TEMRA. Our results revealed a significant increase in the proportions of CD4^+^ Tcm and CD8^+^ Tcm in the patient group, whereas CD4^+^ Tem and CD4^+^ TEMRA cells were markedly decreased. CD4^+^ Tcm, or central memory T helper cells, are a subset of memory T cells that arise from antigen-experienced CD4^+^ T cells. They exhibit distinct homing properties, robust proliferative capacity, and potent immunological responses, representing an intermediate state between naive T cells (Tn) and effector memory T cells (Tem). Notably, previous studies assessing progression risk in SMM using circulating tumor cells (CTCs) have identified the proportion of CD4^+^CXCR3^+^ central memory T cells in peripheral blood as a risk factor. A higher frequency of this subset was associated with an increased risk of disease progression (21). This observation is partially consistent with our findings. Although we observed an increased proportion of CD4^+^ Tcm cells in the bone marrow of MM patients, the functional significance of this alteration remains unclear and requires further investigation. CD8^+^ Tcm cells are part of the “memory pool” of antigen-specific CD8^+^ T cells that remain after the clearance of a primary infection. These cells have the capacity for long-term survival and can rapidly expand upon re-encounter with the same pathogen, differentiating into potent cytotoxic effector T cells (Teff) to effectively control secondary infections (22). In our patient cohort, the increased frequency of this subset may reflect alterations in the immune composition of the bone marrow microenvironment in MM patients. However, the immunological implications of this finding remain to be clarified. In tumors treated with programmed cell death protein 1 (PD-1) blockade, CD4^+^ effector memory T cells (Tem) were significantly reduced in non-responding cell populations, whereas this cell subset increased in responding cells. In contrast, our study results show a significant decrease in the proportion of CD4^+^Tem cells in the patient group (23). Our study showed a decreased proportion of CD4^+^ Tem cells in MM patients. Although these findings may indicate differences in immune states within the tumor microenvironment, the relationship between these phenotypic changes and responses to immunotherapy in MM remains uncertain. CD4^+^TEMRA (T effector memory re-expressed antigen) cells refer to a subset of CD4^+^ T cells that have differentiated into effector cells and re-expressed certain markers after previously encountering an antigen. Previous studies have demonstrated that CD4^+^ TEMRA cells play an important role in the immune response to viral infections (24). In our dataset, the reduced proportion of this subset may reflect alterations in T-cell differentiation within the bone marrow immune microenvironment. Further studies are required to determine whether these changes have functional consequences.

In subsequent analyses, we further evaluated the expression of activation markers CD95, CD278, and HLA-DR across various CD4^+^ and CD8^+^ T-cell subsets. Compared with healthy controls, multiple myeloma patients exhibited a significant increase in the proportions of CD95^+^CD4^-^TEMRA, CD95^+^CD8^-^Tn, CD278^+^CD4^-^TEMRA, and HLA-DR^+^CD4^-^Tem cells. In contrast, the frequencies of CD95^+^CD8^-^Tcm, CD278^+^CD8^-^Tn, CD278^+^CD8^-^Tcm, and HLA-DR^+^CD8^-^Tcm subsets were significantly decreased in the patient group. Activation markers such as CD95, CD278, and HLA-DR are commonly used to characterize the activation status of T cells and may reflect immune activation or chronic immune stimulation. For instance, in the context of CAR-T therapy for relapsed or refractory multiple myeloma, elevated expression of these activation molecules in specific T-cell subsets has been associated with reduced responsiveness to treatment. However, the expression of these markers alone does not necessarily indicate functional competence or dysfunction of T cells. Therefore, these findings should be interpreted as phenotypic observations rather than direct evidence of specific immune mechanisms.

It should be noted that classical T-cell exhaustion markers, such as PD-1, TIM-3, TIGIT, and LAG-3, were not evaluated in this study. Therefore, the current data do not allow direct assessment of T-cell exhaustion or immune dysfunction. While we observed altered expression of activation markers including CD95, CD278, and HLA-DR across various T-cell subsets, these markers alone cannot be interpreted as indicators of functional competence or exhaustion. Accordingly, the findings reported here should be considered phenotypic observations of T-cell subsets within the bone marrow immune microenvironment rather than direct evidence of immune escape or therapeutic response. Future studies incorporating functional assays and exhaustion marker profiling will be essential to clarify the functional implications of these phenotypic changes. Furthermore, we note that measuring activation markers (CD95, ICOS, HLA-DR) alone does not show T-cell function. Functional tests such as cytokine production, Ki-67 proliferation, or cytotoxicity were not done in this study. Therefore, the changes we observed in T-cell subsets only describe their phenotype and cannot confirm activation or dysfunction. Future work with functional assays is needed to understand the biological significance of these changes.

In this study, we analyzed the expression patterns of immune activation markers across different T-cell subsets in real-world multiple myeloma patients. These findings provide a descriptive overview of the immune landscape of T-cell subsets in the bone marrow of MM patients. However, due to limitations in sample size and the lack of additional immunological and cellular biology experiments to validate the results, our conclusions should be interpreted with caution. Moreover, given the considerable heterogeneity among multiple myeloma patients, long-term follow-up, expansion of the patient cohort, and thorough understanding of treatment histories under well-controlled observational conditions are essential to derive more robust and clinically relevant conclusions.

In summary, our study reveals the immune activation status of various T-cell subsets within the immune microenvironment of multiple myeloma patients in real-world settings. These observations provide preliminary insights into the immune characteristics of the MM bone marrow microenvironment and may serve as a basis for future studies aimed at elucidating the functional and clinical implications of these immune alterations.

## Data Availability

The original contributions presented in the study are included in the article/**Supplementary Material**. Further inquiries can be directed to the corresponding author.
