# Supplementary material for: Differential expression of immune activation markers in T cells from patients with multiple myeloma
Source: Front Oncol. 2026 May 20;16:1788729. doi: 10.3389/fonc.2026.1788729 (PMC13229971; doi:10.3389/fonc.2026.1788729)
Supplement: Supplementary file 1 [file DataSheet1.pdf]

**Supplementary Table 1. Spearman correlation analysis of $CD4^+$ and $CD8^+$ T-cell subsets across different R-ISS stages.**

| Cell cluster | $\rho_{I\&II\ vs III}$ | *P*-value | Adjusted *P* |
|---|---|---|---|
| $CD4^+$Tn ($CD45RA^+CD197^+$) | 0.231 | 0.3572 | 1 |
| $CD4^+$Tcm (CD45RA-$CD197^+$) | -0.242 | 0.3338 | 1 |
| $CD4^+$Tem (CD45RA-CD197-) | 0.187 | 0.4582 | 1 |
| $CD4^+$TEMRA ($CD45RA^+$CD197-) | 0.165 | 0.5134 | 1 |
| $CD8^+$ Tn ($CD45RA^+CD197^+$) | 0.187 | 0.4582 | 1 |
| $CD8^+$Tcm (CD45RA-$CD197^+$) | -0.033 | 0.8967 | 1 |
| $CD8^+$Tem (CD45RA-CD197-) | -0.494 | 0.0371 | 0.2968 |
| $CD8^+$TEMRA($CD45RA^+$CD197-) | 0.56 | 0.0156 | 0.1248 |

**Supplementary Table 2. Mann–Whitney U test analysis of $CD4^+$ and $CD8^+$ T-cell subsets across different R-ISS stages.**

| Cell cluster | RISS I&II（%） | RISS III（%） | *P*-value | Adjusted *P* |
|---|---|---|---|---|
| $CD4^+$Tn ($CD45RA^+CD197^+$) | 12.20 ± 10.53 | 14.70 ± 9.49 | 0.608 | 1 |
| $CD4^+$Tcm ($CD45RA$-$CD197^+$) | 55.69 ± 7.83 | 46.44 ± 16.62 | 0.190 | 1 |
| $CD4^+$Tem (CD45RA-CD197-) | 31.63 ± 9.89 | 35.15 ± 9.68 | 0.467 | 1 |
| $CD4^+$TEMRA ($CD45RA^+$CD197-) | 0.50 ± 0.42 | 3.72 ± 10.58 | 0.437 | 1 |
| $CD8^+$ Tn ($CD45RA^+CD197^+$) | 4.41 ± 5.76 | 5.99 ± 8.48 | 0.671 | 1 |
| $CD8^+$Tcm ($CD45RA$-$CD197^+$) | 9.25 ± 10.15 | 6.19 ± 3.73 | 0.371 | 1 |
| $CD8^+$Tem (CD45RA-CD197-) | 75.11 ± 15.92 | 60.45 ± 12.43 | 0.044 | 0.352 |
| $CD8^+$TEMRA($CD45RA^+$CD197-) | 11.18 ± 11.88 | 27.38 ± 12.22 | 0.014 | 0.112 |
